# Molecular characterization of methicillin-resistant *Staphylococcus aureus* clinical strains from the endotracheal tubes of patients with nosocomial pneumonia

**DOI:** 10.1186/s13756-020-0679-z

**Published:** 2020-02-28

**Authors:** Roberto Cabrera, Laia Fernández-Barat, Anna Motos, Rubén López-Aladid, Nil Vázquez, Mauro Panigada, Francisco Álvarez-Lerma, Yuly López, Laura Muñoz, Pedro Castro, Jordi Vila, Antoni Torres

**Affiliations:** 1 Cellex Laboratory, CibeRes (Center for net Biomedical Research Respiratory diseases, 06/06/0028)- Institut d’Investigacions Biomèdiques August Pi i Sunyer (IDIBAPS), School of Medicine, University of Barcelona, Barcelona, Spain; 2 Respiratory Intensive Care Unit, Pulmonology Department, Hospital Clínic, Barcelona, Spain; 3 Department of Anesthesiology, Intensive Care and Emergency, U.O.C. Rianimazione e Terapia Intensiva, Fondazione IRCCS Ca’ Granda, Policlinic Milan, Milan, Italy; 4 Critical Care Department, Hospital del Mar, Critical Illness Research Group (GREPAC), Hospital del Mar Medical Research Institute (IMIM), Barcelona, Spain; 5 Barcelona Global Health Institute, Department of Clinical Microbiology, Hospital Clinic, University of Barcelona, Barcelona, Spain; 6 Internal Medicine Intensive Care Unit, Hospital Clínic, Barcelona, Spain

**Keywords:** Endotracheal tube, Biofilm, Methicillin-resistant *Staphylococcus aureus*, Respiratory infection, Hospital-acquired pneumonia, Ventilator-associated pneumonia, MLST, Mechanism of resistance, Clonal complexes

## Abstract

**Background:**

Among all cases of nosocomial pneumonia, *Staphylococcus aureus* is the second most prevalent pathogen (17.8%). In Europe, 29.9% of the isolates are oxacillin-resistant**.** The changing epidemiology of methicillin-resistant *Staphylococcus aureus* (MRSA) nosocomial infections and the decreasing susceptibility to first-line antibiotics leave clinicians with few therapeutic options. The objective of our study was to determine the antimicrobial susceptibility, the associated molecular mechanisms of resistance and the epidemiological relatedness of MRSA strains isolated from the endotracheal tubes (ETT) of intubated critically ill patients in the intensive care unit (ICU) with nosocomial pneumonia caused by *Staphylococcus aureus*.

**Methods:**

The antimicrobial susceptibility to vancomycin, linezolid, ciprofloxacin, clindamycin, erythromycin, chloramphenicol, fusidic acid, gentamicin, quinupristin-dalfopristin, rifampicin, sulfamethoxazole/trimethoprim, and tetracycline were measured. Resistance mechanisms were then analyzed by polymerase chain reaction and sequencing. Molecular epidemiology was carried out by multi-locus sequence typing.

**Results:**

*S. aureus* isolates were resistant to ciprofloxacin, erythromycin, gentamicin, tetracycline, clindamycin, and fusidic acid. The most frequent mutations in quinolone-resistant *S. aureus* strains were S84L in the *gyrA* gene, V511A in the *gyrB* gene, S144P in the *grlA* gene, and K401R/E in the *grlB* gene. Strains resistant to erythromycin carried the *ermC*, *ermA,* and *msrA* genes; the same *ermC* and *ermA* genes were detected in strains resistant to clindamycin. The *aac(6′)-aph(2″)* gene was related to gentamicin resistance, while resistance to tetracycline was related to *tetK* (efflux pump). The *fusB* gene was detected in the strain resistant to fusidic acid. The most frequent sequence types were ST22, ST8, and ST217, which were distributed in four clonal complexes (CC5, CC22, CC45, and CC59).

**Conclusions:**

High levels of resistance to second-line antimicrobials threatens the treatment of nosocomial respiratory infections due to methicillin-resistant *S. aureus* with decreased susceptibility to linezolid and vancomycin. The wide genotypic diversity found reinforces the central role of ICU infection control in preventing nosocomial transmission.

## Background

Hospital-acquired pneumonia (HAP) and ventilator-associated pneumonia (VAP) are the principal causes of infection among critically ill patients in intensive care units (ICU) [1]. Among all such cases of nosocomial pneumonia, *Staphylococcus aureus* is the second most prevalent pathogen (17.8%), with 29.9% of the isolates in Europe being oxacillin-resistant [2]. The changing epidemiology of methicillin-resistant *Staphylococcus aureus* (MRSA) nosocomial infections, the decreasing susceptibility to first-line antibiotics, such as vancomycin-intermediate *Staphylococcus aureus* (VISA), linezolid resistant MRSA [3], and community-associated MRSA (CA-MRSA) leave clinicians with few therapeutic options. In this context, an accurate description of the mechanisms of antimicrobial resistance in MRSA nosocomial pneumonia in ICU could help in the design of novel therapies. Knowledge of resistance-related phenotypic and genotypic changes is critical for the development of new drugs. When designing a new antibiotic, the previously described resistance mechanisms must be taken into account. The new antimicrobial should be able to overcome the resistance mechanisms, or should be aimed at new targets where the probability that the microorganism has developed resistance is lower [4].

Given that few new antimicrobial agents have been approved in the last 10 years, it is anticipated that the problems associated with resistance will only worsen. Antibiotics currently approved for MRSA nosocomial pneumonia are linezolid (an oxazolidinone), vancomycin (a glycopeptide), ceftobiprole (an extended-spectrum cephalosporin) and Telavancin (a lipoglycopeptide). Tedizolid (a second-generation oxazolidinone) is pending authorization for systemic treatment of HAP [5]. Other secondary options when these agents cannot be used include, either alone or in combination, quinolones (ciprofloxacin or levofloxacin), macrolides (erythromycin), aminoglycosides (gentamicin), tetracyclines, clindamycin (a lincosamide), and fusidic acid.

A wide range of resistance mechanisms have been described for *S. aureus* including PBP alterations (β-lactam agents), cell wall structure modifications (glycopeptides), point mutations in the quinolone resistance-determining regions of GyrA and GrlA (quinolones), inactivating enzymes (aminoglycosides) ribosome alterations (macrolides, lincosamides, oxazolidones and tetracyclines), efflux pumps (tetracyclines, macrolides, quinolones) or spontaneus mutations in the gene *fusA* encoding the ribosomal translocase elongation factor G (fusidic acid) [6, 7]. However, little recent information is available on the mechanisms of resistance in *S. aureus* strains obtained from mechanically ventilated patients, or whether or not these mechanisms are associated with particular circulating *S. aureus* clones.

The aim of this study was to determine the antimicrobial susceptibility, the associated molecular mechanisms of resistance, and the epidemiological relatedness of MRSA strains isolated from the ETTs of intubated critically ill patients in the intensive care unit (ICU) with nosocomial pneumonia caused by *Staphylococcus aureus*.

## Materials and methods

### Study design, sample collection and bacterial isolates

Clinical *S. aureus* (17 MRSA and three methicillin-susceptible isolates) were collected from ETTs after extubation during a prospective observational study carried out in four European tertiary hospitals from September 2013 to December 2016 [8]. The participating centers were the Hospital Clinic of Barcelona (Spain), the Hospital del Mar (Critical Care Department; Barcelona, Spain), the Hospital Universitario Central de Asturias (Intensive Medicine Service; Oviedo, Spain), and the Fondazione IRCCS Ca′ Granda (Adult Intensive Care; Ospedale Maggiore Policlinico, Milan, Italy). Patients were included if they were older than 18 years, mechanically ventilated (with ≥48 h of orotracheal intubation), had microbiologically confirmed nosocomial MRSA pneumonia, and were treated for ≥48 h with either linezolid or vancomycin.

This study was carried out in compliance with the latest revision of the Declaration of Helsinki (Fortaleza, Brazil, October 2013) and was conducted in accordance with the requirements of Law 14/2007 of July 3, of Biomedical Research. The study was approved by the institution’s Internal Review Board (registry number 2012/7927). Written informed consent was obtained from patients or their next-of-kin.

### Antimicrobial susceptibility testing

The minimal inhibitory concentrations of vancomycin and linezolid were determined by E-Test. Antimicrobial susceptibility was performed using the Kirby-Bauer method and the ATCC 25923 strain (*S. aureus*) as a control [9]. The following antibiotics were tested: ciprofloxacin (5 μg), clindamycin (2 μg), erythromycin (15 μg), chloramphenicol (30 μg), fusidic acid (10 μg), gentamicin (10 μg), quinupristin-dalfopristin (15 μg), rifampicin (5 μg), Sulfamethoxazole/trimethoprim (25 μg), and tetracycline (30 μg). Screening of inducible clindamycin resistance was performed by the D-test for strains resistant to clindamycin. Replicates of each susceptibility test were performed. All results were interpreted according to the criteria of the European Committee on Antimicrobial Susceptibility Testing (EUCAST) [10].

### Mechanisms of resistance

We tested the most common mechanisms of resistance to ciprofloxacin, clindamycin, erythromycin, chloramphenicol, fusidic acid, gentamicin, quinupristin-dalfopristin, rifampicin, sulfamethoxazole/trimethoprim, and tetracycline. Each mechanism was screened by polymerase chain reaction (PCR) with the primers and conditions shown in Table 1 and electrophoresis in agarose gel using the 100-bp DNA ladder as a marker for molecular weight (Invitrogen). The PCR products were sequenced by Sanger methods (Genewiz, Germany), and were analyzed by alignment with the template sequence at GenBank [7].

**Table 1:** Primers used in this study

Primer Pair	Amplified product	Sequence (5′ to 3′)	Amplicom size	Anneling temperature	References
gyrA-F	gyrA	ATG GCT GAA TTA CCT CAA TC	398 bp	55°C	13
gyrA-R		GTG TGA TTT TAG TCA TAC GC			
gyrB-F	gyrB	CAGCGTTAGATGTAGCAAGC	680 bp	55°C	17
gyrB-R		CGATTTTGTGATATCTTGCTTTCG			
grlA-F	grlA	CAG TCG GTG ATG TTA TTG GT	469 bp	55°C	13
grlA-R		CCT TGA ATA ATA CCA CCA GT			
grlB-F	grlB	GIG AAG CIG CAC GTA A	363 bp	50°C	13
grlB-R		TCI GTA TCI GCA TCA GTC AT			
ermA-F	erm(A)	TAT CTT ATC GTT GAG AAG GGA TT	138 bp	55°C	5
ermA-R		CTA CAC TTG GCT TAG GAT GAA A			
ermC-F	erm(C)	CTT GTT GAT CAC GAT AAT TTC C	189 bp	55°C	5
ermCR		ATC TTT TAG CAA ACC CGT ATT C			
msrA-F	msrA	TCC AAT CAT TGC ACA AAA TC	162 bp	55°C	5
msrA-R		AAT TCC CTC TAT TTG GTG GT			
aac(6′)-aph(2″)F	aac(6′)-aph(2″)	TTG GGA AGA TGA AGT TTT TAG A	173 bp	55°C	5
aac(6′)-aph(2″)R		CCT TTA CTC CAA TAA TTT GGC T			
tetK-F	tetK	GTA GCG ACA ATA GGT AAT AGT	360 bp	55°C	6
tetK-R		GTA GTG ACA ATA AAC CTC CTA			
fusB-F	fusB	ATT CAA TCG GAA AAC TAT AAT GAT A	292 bp	60°C	21
fusB-R		TTA TAT ATT TCC GAT TTG ATG CAA G			
16srRNA-F	16S rRNA	GGA GGA AGG TGG GGA TGA CG	245 bp	55°C	5
16srRNA-R		ATG GTG TGA CGG GCG GTG TG			
arcC-F	arcC	TTGATTCACCAGCGCGTATTGTC	450 bp	55°C	10
arcC-R		AGGTATCTGCTTCAATCAGCG			
aroE-F	aroE	ATCGGAAATCCTATTTCACATTC	450 bp	55°C	10
aroE-R		GGTGTTGTATTAATAACGATATC			
glpF-F	glpF	CTAGGAACTGCAATCTTAATCC	450 bp	55°C	10
glpF-R		TGGTAAAATCGCATGTCCAATTC			
gmk-F	gmk	ATCGTTTTATCGGGACCATC	450 bp	55°C	10
gmk-R		TCATTAACTACAACGTAATCGTA			
pta-F	pta	GTTAAAATCGTATTACCTGAAGG	450 bp	55°C	10
pta-R		GACCCTTTTGTTGAAAAGCTTAA			
tpi-F	tpi	TCGTTCATTCTGAACGTCGTGAA	450 bp	55°C	10
tpi-R		TTTGCACCTTCTAACAATTGTAC			
yqiL-F	yqiL	CAGCATACAGGACACCTATTGGC	450 bp	55°C	10
yqiL-R		CGTTGAGGAATCGATACTGGAAC			

### Multi-locus sequence typing

Allelic profiles of seven *S. aureus* housekeeping genes (*arcC, aroE, glpF, gmk, pta, tpi, yqiL*) were analyzed and confirmed in 2% agarose gel. Next, PCR products were sequenced by Gemewiz and sequence alignment was done by the ClustalW software. These genes were linked by the multi-locus sequence typing (MLST) database (https://MLST.net; https://pubmlst.org/saureus/) to assign the sequence type. Phylogenetic analysis was carried out using comparative eBURST V3 software employing the eBURST algorithm (http://www.phyloviz.net/goeburst) [11, 12].

## Results

### MRSA positive samples

Twenty strains of *S. aureus* were isolated and characterized. Of these, 17 were methicillin-resistant, as confirmed by the oxacillin E-Test, and three were methicillin- susceptible.

### Antimicrobial susceptibility

**Fig. 1:**
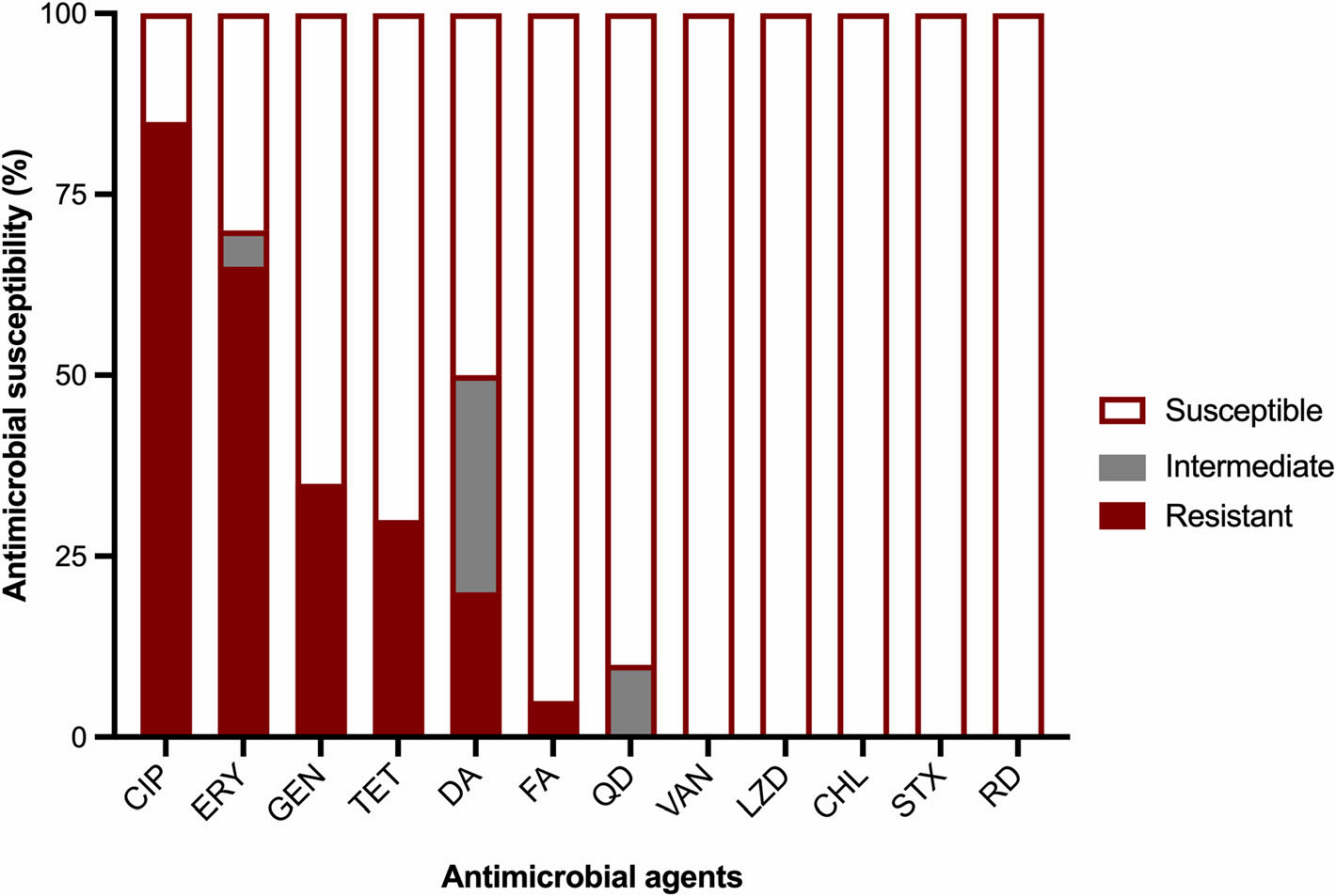
Antimicrobial susceptibility. Abbreviations: CIP, ciprofloxacin; ERY, erythromycin; GEN, gentamicin; TET, teracycline; DA, clindamycin; FA, fusidic acid; QD, quinupristin-dalfopristin; VAN, vancomycin; LZD, linezolid; CHL, cloranphenicol; STX, sulfamethoxazole/trimetoprim; RD, rifampicin

Although there was high susceptibility to linezolid, three strains showed hetero-resistant subpopulations to this antimicrobial agent (strain 1: CC22 (HUCA), strain 2: CC59 (HCP) and strain 8: CC22 (Hospital del Mar) (Table 2). In total, 40% of *S. aureus* strains were resistant to three or more different antimicrobial agents, with 85% resistant to ciprofloxacin, 65% to erythromycin, 35% to gentamicin, 30% to tetracycline, 20% to clindamycin, and 5% to fusidic acid (Fig. 1). Two strains showed inducible resistance to clindamycin. All the strains were susceptible to vancomycin, linezolid, chloramphenicol, sulfamethoxazole/trimethoprim and rifampicin.

**Table 2:** Resistance patterns and mechanisms of resistance

					QRDR Mutations				Resistant genes			
ETT code	STs	CC	Origin	Resistance patern	gyrA	gyrB	grlA	grlB	ERY- DA	GEN	TET	FA
1	ST954	CC22	HUCA	CIP - ERY- DA			S144P	K401R	*ermC*			
2	ST87	CC59	HCP	ERY- TET					*msrA*		*TeT K*	
3	*ST8	CC5	HCP	CIP - GEN - TET	S84L, T129I		S144P	K401R		*aac(6´)-aph(2")*	*TeT K*	
4	ST217	CC22	HCP	CIP - ERY	S84L, S85P		S144P, V82S, Y83V, E84R	K401R, D507E	*ermC*			
5	*ST8	CC5	HCP	ERY - TET					*msrA*		*TeT K*	
6	ST45	CC45	HCP	CIP - GEN	E134N, L135T		S144P	K401E		*aac(6´)-aph(2")*		
7	ST22	CC22	Hospital del Mar	CIP - ERY - DA	S84L, T129K	Y500T, H501T	S144P, G78A, E84R		*ermC*			
8	ST22	CC22	Hospital del Mar	CIP - ERY	S84L, S85P, T129I	V511A, V303C	S144P, S81P		*ermC*			
9	ST8	CC5	Hospital del Mar	CIP - ERY - GEN - TET	S84L, T129K, I131S		S144P	K401E	*msrA*	*aac(6´)-aph(2")*	*TeT K*	
10	ST22	CC22	Hospital del Mar	CIP	S84L, S85P	V511A, R447H	S144P	K401R, L440F				
11	ST22	CC22	Hospital del Mar	CIP - GEN - TET	S84L, K130G	V511A, G339D, K312T	S144P	K401E, K400Q, D507E		*aac(6´)-aph(2")*	*TeT K*	
12	ST22	CC22	Policlinic Milan	CIP - ERY	S84L, T129K		S144P	K401R	*ermC*			
13	ST1535	-	Policlinic Milan	CIP - GEN - TET - FA		R447L	S144P	K403Q, H478Y, D507E		*aac(6´)-aph(2")*	*TeT K*	*FusB*
14	ST22	CC22	Policlinic Milan	CIP - ERY	S84L, T129I	H501T	S144P, S80F		*ermC*			
15	ST83	CC5	Policlinic Milan	CIP - ERY	S84L	H501T	S144P		*ermC*			
16	ST217	CC22	Policlinic Milan	CIP - ERY - GEN - DA	S84L	L418F	S144P	K401E	*ermA*	*aac(6´)-aph(2")*		
17	ST403	CC5	Policlinic Milan	CIP	T129K	V511A, S425G, R447N	S144P	D503E, A504P				
18	ST1221	CC5	Policlinic Milan	CIP - ERY	S84L, E88A		S144P, S80F	K401E, D507N	*ermC*			
19	ST22	CC22	Policlinic Milan	CIP - ERY - GEN - DA	S84L		S144P	K403Q, A504P, D507E	*ermA*	*aac(6´)-aph(2")*		

* ETT collected at different ICU. ETT code 3, 5, and 6 = Methicillin sensitive *Staphylococcus aureus*

*Abbreviations*: *CC* Clonal Complex, *CIP* Ciprofloxacin, *DA* Clindamycin, *ERY* Erythromycin, *FA* Fusidic acid, *GEN* Gentamicin, *HCP* Hospital Clinic (Spain), *HUCA* Hospital Universitario Central de Asturias (Spain); Policlinic Milan, Fondazione IRCCS Ca′ Granda (Italy), *QRDR* Quinolone Resistance-Determining Region, *ST* sequence type; Strain 20, susceptible to all antimicrobial agents tested (ST217, CC22), *TET* Tetracycline

### Mechanisms of resistance

Mutations in *gyrA, gyrB*, *grlA*, and *grlB* genes were found in ciprofloxacin-resistant *S. aureus* strains. The most frequent mutations were S84 L in *gyrA* (76.5%), V511A in *gyrB* (23.5%), S144P in *grlA* (100%), and K401R/E in *grlB* (58.8%). Erythromycin resistance was related to the *ermC* (61.5%), *ermA* (15.4%), and *msrA* genes (23.1%). Aminoglycoside-resistant strains contained the *aac(6′)/aph(2″)* gene, while tetracycline-resistant strains contained the *tetK* gene. In strains that were resistant to clindamycin, *the ermC* (50%) and *ermA* (50%) were detected in equal numbers. Finally, the *fusB* gene was detected in the strain resistant to fusidic acid. The presence of the *fusB* gene in plasmid pUB101 and plasmid pUB102 was not confirmed.

### Phylogenetic analysis

**Fig. 2:**
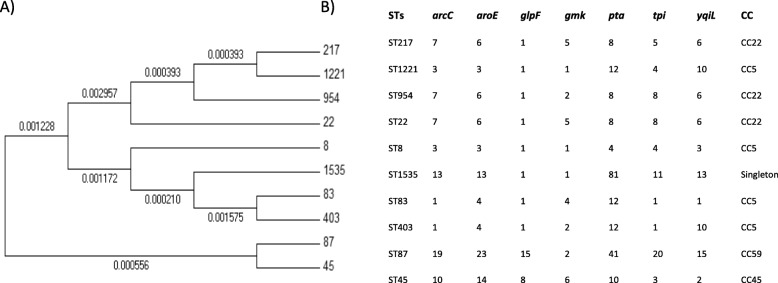
Phylogenetic tree of the different sequence types and their corresponding clonal complexes. **a** Phylogenetic tree of all sequence types found in the isolated MRSA strains. **b** Sequence types, alleles for the different housekeeping genes (per sequence type), and clonal complexes where included. The included genes are as follows: *arcC* (carbamate kinase), *aroE* (shikimate dehydrogenase), *glpF* (glycerol kinase), *gmK* (guanylate kinase), *pta* (phosphate acetyltransferase), *tpi* (triosephosphate isomerase), *yqi*l (acetyl coenzyme A acetyltransferase)

**Fig. 3:**
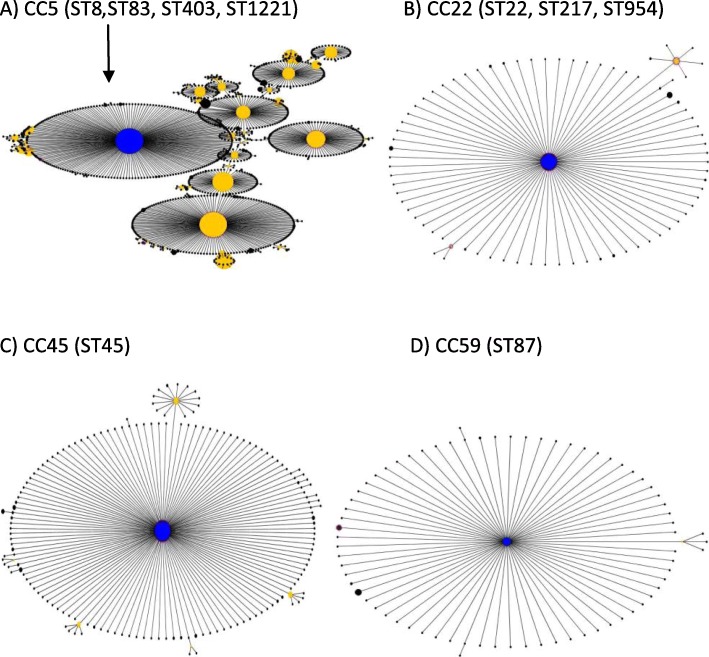
Clonal complexes where the strains are located. **a** CC5, where the founder is ST5. Within this complex, we find ST8, (Strains 3, 5, and 9), ST83 (Strain 15), ST403 (Strain 17), and ST1221 (Strain 18). **b** CC22, where the founder is ST22 (Strains 7, 8, 10, 11, 12, 14, and 19). Within this complex, we also found ST217 (Strains 4, 16, and 20) and ST954 (Strain 1). **c** CC45, where ST45 (Strain 6) is its founder. **d** CC59 was predicted from ST87 (Strain 2) and ST59. Finally, ST1535 (Strain 13) did not belong to any clonal complex and was recorded as a singleton. Abbreviations: CC, clonal complex; MRSA, methicillin resistant *Staphylococcus aureus*; ST, sequence types

The following *S. aureus* sequence types (STs) were the most common: ST22 (35%), ST8 (15%), and ST217 (15%). However, ST87, ST83, ST45, ST954, ST403, ST1221, and ST1535 were found with a frequency of 5%. The hospitals where these were collected are shown in Table 2, and the phylogenetic tree shows the genetic proximity (Fig. 2). The allelic profile of each sequence type and clonal complex is also shown in Fig. 2. Our sequence types were distributed in four clonal complexes: CC5 included ST8, ST83, ST403 and ST1221; CC22 included ST22, ST217, and ST954; CC45 included only ST45; and CC59 included only ST87 (Fig. 3). In addition, ST1535 was distributed as a singleton. The strains isolated at the Hospital Clínic were distributed in the four clonal complexes, while the strains at the Hospital del Mar and at the Hospital of Milan were distributed in clonal complexes CC5 and CC22. The most frequent clonal complexes were CC22 and CC5, which accounted for 55 and 30% of local MRSA strains respectively.

## Discussion

The present study reports several important findings regarding the antimicrobial susceptibility, resistance mechanisms, sequence type distributions, and clonality of MRSA strains obtained from ICU respiratory infections in Spain and Italy. At the participating ICUs, *S. aureus* was not found to be resistant to first-line antibiotics such as linezolid and vancomycin. Although the prevalence of MRSA in the participating centres was low, the mechanisms of resistance described may also be representative for sites with high MRSA prevalence because the MRSA collected during this study corresponded to highly disseminated clonal complexes (CC22 and CC5). In addition, these strains harbored a wide range of antimicrobial mechanisms to second-line antibiotics, including ciprofloxacin, erythromycin, gentamicin, tetracycline, clindamycin, and fusidic acid.

Although our strains did not show resistance to linezolid, the detection of subpopulations resistant to this antimicrobial is a finding that merits comment. Hetero-resistance (HR) is an unstable phenomenon with a high incidence in several bacterial strains, according to recent reports. It is considered unstable because subpopulations defined as hetero-resistant in one susceptibility test may no longer appear as such if the test is repeated. The recent finding of plasmid-associated HR mechanisms emphasizes the problem, since these HR mechanisms may spread horizontally between pathogens. The lack of routine determination by many laboratories and the decrease in antimicrobial activity may have clinical implications. Another important feature is that the decrease in antimicrobial activity caused by this phenomenon is not reflected in the MIC value. Previous research also suggests that HR can indeed be responsible for treatment failure in *S. aureus* infections. However, this phenomenon is not detected by established procedures and new methods are needed for rapid identification of HR in pathogenic bacteria [13].

Few reports on MRSA resistance mechanisms contain an exhaustive evaluation of antibiotics. In our study we often identified a combination of several resistance mechanisms for MRSA strains, such as spontaneous mutations that decrease bacterial replication, transferable and chromosomal efflux pumps, and antimicrobial or target-modifying enzymes. In terms of antimicrobial susceptibility, similar results have been reported in other studies. For instance, in a series of MRSA strains isolated from different samples, Kitti et al. [14] found high levels of resistance to ciprofloxacin (72.1%), erythromycin (86.9%), gentamicin (72.1%), and clindamycin (86.9%).

In agreement with Sierra et al. [15], we found mutations in the quinolone resistance-determining regions (*gyrA, grlA, gyrB, grlB*) of ciprofloxacin-resistant *S. aureus* strains. Several reports have indicated that topoisomerase IV is the primary target for quinolone resistance in Gram-positive microorganisms, including *S. aureus* with DNA-gyrase acting as secondary target, with specific point mutations at GrlA (subunit A of the topoisomerase IV) and GyrA (subunit A of the DNA-gyrase) as the most relevant [16]. However, our results differ because the mutation S144P in *grlA* gene may be a polymorphism, given that it is found in both susceptible and resistant strains. This means that the primary target in our strains is the DNA-gyrase. Studies in Japan have not shown mutations in the *gyrB* and *grlB* genes [17], which in any case tend to be infrequent in MRSA strains. Nevertheless, some of our strains showed more than three mutation points in each gene. Therefore, further studies are needed to confirm whether these mutations determine resistance or genetic polymorphisms. In addition, in *gyrA* and *grlA*, some rare mutations were described (Table 2). Because antibiotic combinations are used during nosocomial pneumonia treatment, these strains are exposed to strong antibiotic selection pressure which may contribute to the high number of mutations found here compared with prior studies. For instance, it has been demonstrated that hospital-acquired MRSA harbors higher levels of antimicrobial resistance than community-acquired MRSA [18].

Analyzing 206 strains of *S. aureus* from different centers in Canada, China, and France, Martineau et al. attributed erythromycin resistance to *ermA* (98%), *ermB* (21%), *ermC* (2.4%), and *msrA* (1%) genes. In our study, erythromycin resistance in *S. aureus* was mediated by the *ermA* (15%), *ermC* (62%) and *msrA* (23%) genes. In an Algerian study, erythromycin resistance was much lower (37.8%) than in our study (65%). However, the authors of the Algerian study included *S. aureus* from food, nosocomial, and community-acquired infections and identified only the *ermC* gene [6, 19]; the fact that we only isolated strains from nosocomial pneumonia, whereas the other studies used different sources, could explain the differences observed.

Similarly, Yilmaz et al. also included *S. aureus* from different clinical samples and found the *ermC* and *ermA* genes in strains with resistance to clindamycin [20]. They reported a lower prevalence (6%) of resistance to clindamycin compared to ours (20%), and detected the *lnuA* gene instead of the *erm* genes [20, 21]. Some *S. aureus* strains have shown inducible resistance to clindamycin after exposure to subinhibitory concentrations of erythromycin [22]. In our study, the D-test [10] revealed two MRSA isolates with inducible resistance to clindamycin. This finding is important because clindamycin is used in the treatment not only of pneumonia but also of muscle, bone, skin, and soft tissue infection.

In our study, gentamicin resistance was related to the *aac(6′)/aph(2′′)*. Choi et al. detected higher proportions of the *aac(6′)/aph(2′′)* gene in MRSA isolates from blood, sputum, urine, and pus samples (65%) than we did in ETT specimens (35%), but they also found prevalences of *ant(4′)-Ia* and *aph(3′)-IIIa* of 41 and 9% respectively [23]. Yilmaz et al. found that four of six MRSA isolates carried the same *aac(6′)/aph(2′′)* gene*.* Nevertheless, our findings are consistent with those of Martineau et al., who observed a higher number of *S. aureus* isolates, among which all those with gentamicin resistance had the *aac(6′)/aph(2′′)* gene [6].

Tetracycline resistance in *S. aureus* at our ICU was mediated only by the *tetK* gene. By contrast, other studies have found different proportions of involvement of the *tetK* or *tetM* genes alone or in combination; for instance, Strommenger et al. identified ten strains of *S. aureus* carrying *tetK*, *tetM*, or both genes [7]. Yilmaz et al. identified nine strains of *S. aureus* with the *tetM* gene and ten with the *tetK* gene [20]. Finally, Achek et al. detected both the *tetK* and *tetM* genes in ten *S. aureus* isolates from clinical samples [19].

Although *fusB* was initially thought to be the only gene to encode a protein capable of protecting EF-G, a whole family of related *fusB*-like proteins has since been described. Thus, mutations in two more genes (*fusC* and *fusD*) can lead to staphylococcal resistance to fusidic acid [24]. Several studies have also reported an increase in resistance to fusidic acid. We suggest a chromosomal location of the fusB gene because the primers we used were developed in previous studies by O’Neil et al. in which the fusB gene was detected in total DNA preparations but not in plasmid DNA preparations, indicating a chromosomal location for this resistance determinant (different *fusB* genes have been discovered on plasmid pUB101 and plasmid pUB102). Some previous data suggest that chromosomal *fusB* was associated with epidemic strains of *S. aureus* [25]. Interestingly, in another study *fusB*-type resistance (*fusB* and *fusC*) was found in 87% of MRSA isolates [24], with an association between *fusB* and clonal complexes CC45 and CC97. By contrast, we found only one strain with *fusB*, and this was the singleton ST1535. Fusidic acid is a topical drug that is used for the treatment of staphylococcal skin infections, but its increased use appears to have led to the emergence and dissemination of resistant staphylococci [26].

The molecular epidemiology of MRSA in bloodstream infections has been described previously, but less frequently in respiratory infections contracted in the ICU [27, 28]. CC22 is one of the largest circulating clonal complexes associated with hospital-acquired MRSA in Europe (UK) and Asia (Kuala Lumpur, China) [11, 29], while studies of nosocomial pneumonia indicate that CC5 is associated with MRSA strains originating mostly from the US, Europe (Portugal), Asia (China), Africa (Algeria) and Latin America (Argentina and Chile) [18, 19, 30, 31]. Despite the marked heterogeneity of the sequence types in this study, CC22 and CC5 were the main clonal complexes detected. Specific resistance mechanisms can be associated with clonality, since a higher number of these mechanisms were found in the widely expanded CC5 and CC22 clones than in the CC45 and CC59 clones [32]. Consistent with our results, previous studies (in the US, Portugal and Japan) have found CC5 and CC59 to be associated with extensive multi-drug resistance, but not CC45 [32]. Another important point to stress is that these CCs had previously been associated with virulent *S. aureus* strains.

The heterogeneity of MRSA sequence types at each hospital suggests that ICU cross-transmission has decreased, probably due to the introduction of VAP prevention bundles, isolation measures, and hospital hygiene measures over the last 10 years. Thus, our study indicates that other sources of MRSA transmission such as nasal carriage constitutes risk factors for ICU and nosocomial pneumonia. Although we did not assess nasal MRSA carriage in our study, it has been shown to be an independent risk factor for ICU pneumonia in previous work [33].

This study has some limitations. First, the number of strains is relatively low because *S. aureus* and MRSA are infrequent causes of nosocomial pneumonia in Spain. However, we also included strains from Italy and found a high heterogeneity of sequence types, which may be representative of the current clones circulating as causes of hospital-acquired MRSA in Europe. Second, although we did not assess the virulence of our MRSA strains, some of the clonal complexes identified, such as the CC59 and CC45, have been shown to be closely related to virulent strains. Immune evasion cluster (IEC) genes have been associated with CC59 (IEC-hemolysin genes) and CC45 (IEC-enterotoxin-hemolysin genes) [32].

Despite the limitations mentioned, we think that this study is important for establishing the epidemiology of *S. aureus.* Little recent information is available on the resistance mechanisms of action of *S. aureus* strains obtained from mechanically ventilated patients, and it is unclear whether or not these mechanisms are associated with particular circulating *S. aureus* clones. We also observed the presence of linezolid hetero-resistance and high resistance to second-line antibiotics in MRSA strains isolated from endotracheal tubes in humans mechanically ventilated for long periods in the ICU. These findings show that MRSA infection is still relevant in southern Europe, with a high capacity of resistance to different antimicrobials, an extensive battery of resistance mechanisms, and a wide clonal variability.

## Conclusions

The high level of second-line antimicrobial resistance represents a major problem for the treatment of nosocomial respiratory infections due to MRSA, which display decreased susceptibility to linezolid and vancomycin. Nevertheless, the mechanisms of resistance reported may be useful for the design of new strategies for preventing MRSA. The wide genotypic diversity found reinforces the central role of infection control measures for preventing nosocomial MRSA transmission in the ICU.

## Data Availability

All data generated or analysed during this study are included in this published article.
